# Venous endothelial remodeling mediated by *MARCKS* promotes angiogenesis and tumor progression in hepatocellular carcinoma: insights from single-cell RNA sequencing

**DOI:** 10.3389/fimmu.2026.1734982

**Published:** 2026-03-12

**Authors:** Yutong Zhao, Jihui Huo, Changxuan Wang, Minjin Zhan, Kai Lei, Qi Zhou

**Affiliations:** 1 Department of Oncology, The First Affiliated Hospital of Sun Yat-sen University, Guangzhou, Guangdong, China; 2 Department of Urology, The First Affiliated Hospital, Sun Yat-Sen University, Guangzhou, China; 3 Department of Obstetrics and Gynecology, HuiYa Hospital of The First Affiliated Hospital, Sun Yat-sen University, Huizhou, Guangdong, China; 4 Department of Hepatobiliary Surgery, HuiYa Hospital of The First Affiliated Hospital, Sun Yat-sen University, Huizhou, Guangdong, China; 5 Center of Hepato-Pancreato-Biliary Surgery, The First Affiliated Hospital of Sun Yat-sen University, Guangzhou, Guangdong, China

**Keywords:** angiogenesis, hepatocellular carcinoma, immune microenvironment, *MARCKS*, venous endothelial cells

## Abstract

**Background:**

Hepatocellular carcinoma (HCC) is characterized by pronounced heterogeneity and extensive angiogenesis. However, anti-angiogenic therapies often show limited clinical benefit due to therapeutic resistance. Understanding endothelial cell heterogeneity and identifying key regulators of tumor angiogenesis are therefore essential for improving treatment strategies.

**Methods:**

We integrated multiple single-cell RNA sequencing (scRNA-seq) datasets to systematically characterize endothelial cell heterogeneity in the HCC microenvironment. Based on genes enriched in venous endothelial cells, we developed a prognostic risk model termed the Vein Endothelial-related Risk Scores (VERS). The functional role of the key gene *MARCKS* was further evaluated using *in vitro* assays and *in vivo* xenograft models.

**Results:**

Venous endothelial cells (VenECs) were identified as key initiators of tumor angiogenesis in HCC. Among the VERS genes, *MARCKS* emerges as a robust predictor of poor clinical outcome. Functional assays reveal that *MARCKS* knockdown impairs endothelial cell proliferation, migration and invasion, and attenuates the pro-tumorigenic effects of endothelial-conditioned media. *In vivo*, *MARCKS* silencing significantly suppresses tumor growth and vascularization.

**Discussion:**

Our findings reveal a critical role for venous endothelial cells in HCC angiogenesis and identify *MARCKS* as a potential therapeutic target, providing molecular insights for precision oncology in HCC.

## Introduction

Hepatocellular carcinoma (HCC) accounts for approximately 50% of global cases in China alone, yet the five-year survival rate remains dismal at only ~12.1% (1). Despite progress in surgical resection, liver transplantation, transarterial chemoembolization (TACE) and radiotherapy, long-term outcomes for HCC patients remain unsatisfactory—largely due to the tumor’s high degree of molecular heterogeneity and a profoundly immunosuppressive tumor microenvironment (2–4). These challenges highlight the urgent need for robust biomarkers capable of predicting disease progression and guiding personalized therapeutic strategies.

Endothelial cells (ECs), as a key component of the tumor microenvironment, play essential roles in angiogenesis and HCC progression. Based on their anatomical location within the vascular tree, ECs can be classified into five types of cells (5). Beyond mediating the exchange of gases and metabolites, ECs regulate hemodynamics, coagulation, neovascularization, and inflammation under both physiological and pathological conditions (6). In tumors, neovascularization sustains rapid proliferation by supplying oxygen and nutrients, while simultaneously establishing an immunosuppressive microenvironment (7, 8). This functional heterogeneity of ECs not only underlies their diverse roles in tumor biology but also contributes to the limited efficacy of anti-angiogenic therapies (AATs), as resistance often emerges from inter- and intra-tumoral variability in EC phenotypes across different stages of angiogenesis (7, 9–11). Notably, recent evidence identifies venous ECs (VenECs) as the cellular origin of tumor angiogenesis (7). Targeting this specific endothelial subset may offer a novel therapeutic avenue, particularly for patients unresponsive to conventional AATs, which primarily act on mature vasculature.

In this study, we integrated bulk RNA sequencing data from The Cancer Genome Atlas Liver Hepatocellular Carcinoma (TCGA-LIHC) cohort and multiple independent single-cell RNA-seq datasets (GSE151530, GSE125449, and GSE149614) to systematically characterize the tumor endothelial landscape in HCC. Combining computational deconvolution, survival modeling, and functional validation *in vitro*, we identified *MARCKS* as a key regulator of venous endothelial activation and a novel prognostic biomarker.

## Methods

### Data collection

This study included patients from two independent cohorts: the Liver Hepatocellular Carcinoma (TCGA-LIHC) cohort from The Cancer Genome Atlas and the GSE151530 dataset from the Gene Expression Omnibus (GEO). To ensure clinical reliability and avoid bias from perioperative or non-cancer-related early deaths, we excluded patients with overall survival (OS) time less than 30 days in both cohorts. To address technical heterogeneity between RNA-seq (TCGA) and microarray (GEO) platforms, we first intersected the gene sets and retained only protein-coding genes present in both datasets. Expression matrices for each cohort were then independently Z-score normalized across samples. Subsequently, batch effects across platforms were corrected using the ComBat algorithm implemented in the sva R package (version 3.48.0), with empirical Bayes estimation to preserve biological variation while removing platform-specific artifacts. This harmonized expression matrix served as the input for all downstream analyzes.

### scRNA-seq data preprocessing

Single-cell RNA sequencing data were obtained from three publicly available datasets in the Gene Expression Omnibus (GEO): GSE151530 (12), GSE125449 (13) and GSE149614 (14) comprises scRNA-seq data generated from human HCC samples using the Illumina sequencing platform. Describe in detail, these datasets collectively encompass transcriptomic profiles from 28 patients with histologically confirmed HCC who underwent surgical resection of treatment-naïve primary liver tumors. Specifically, GSE151530 contributed samples from 13 patients, GSE125449 from 5 patients, and GSE149614 from 10 patients. All included individuals met the following criteria: (1) a definitive diagnosis of HCC; (2) surgical removal of the primary tumor without prior systemic or locoregional therapy; and (3) availability of high-quality scRNA-seq data from tumor tissue. Notably, none of the analyzed samples originated from intrahepatic cholangiocarcinoma (ICC), metastatic lymph nodes, portal vein tumor thrombus (PVTT), or non-tumor liver tissue—thus ensuring biological consistency within our integrated cohort. The unique molecular identifier (UMI) count matrix was imported and converted into a Seurat object using the R package “Seurat” (version 4.3.0). Quality control procedures were then applied to remove low-quality cells based on the following criteria: samples with fewer than 500 cells, cells with fewer than 300 detected genes, and cells with a mitochondrial gene content exceeding 15% were excluded. To identify potential doublets, the “DoubletFinder” R package (version 2.0.3) was used. The expected doublet rate was adjusted according to the Poisson doublet formation model, taking cell concentration into account. After completing the quality control steps, the final dataset included 41,301 cells and 25,714 genes, which were retained for subsequent analysis.

### InferCNV-based validation of tumor cell identity through copy number variation profiling

To validate the identity of tumor cells, we performed copy number variation (CNV) profiling using inferCNV (version 1.16.0), a computational tool designed to detect somatic CNVs from single-cell RNA-seq data. Cells were grouped into two populations: Tumor cells and T cells, based on their annotation from Seurat clustering and marker gene expression. The reference cell population was set as T cells, which are expected to have diploid genomes. inferCNV was run with default parameters, including normalization by median expression per cell and log2 transformation of read counts. CNV profiles were visualized as heatmaps across all autosomes (chromosomes 1-22), where red indicates gains and blue indicates losses relative to the reference. The interCNV score (log2 ratio of mean expression in test vs. reference cells) was used to assess chromosomal aberrations.

### Endothelial cell subtype annotation

After initial quality control and normalization, endothelial cells were first identified based on the co-expression of canonical endothelial markers (*PECAM1*, *VWF*, and *CDH5*) and exclusion of hematopoietic (*PTPRC*/*CD45*), epithelial (*EPCAM*), and mesenchymal (*ACTA2*, *PDGFRB*) lineages. The isolated endothelial compartment underwent standard Seurat-based preprocessing, including identification of variable features, scaling, principal component analysis (PCA), and batch effect correction using Harmony with “tissue origin” as a covariate. Clustering was performed on the Harmony-corrected low-dimensional space at a resolution of 0.2 to resolve biologically meaningful subpopulations.

**Figure 1:**
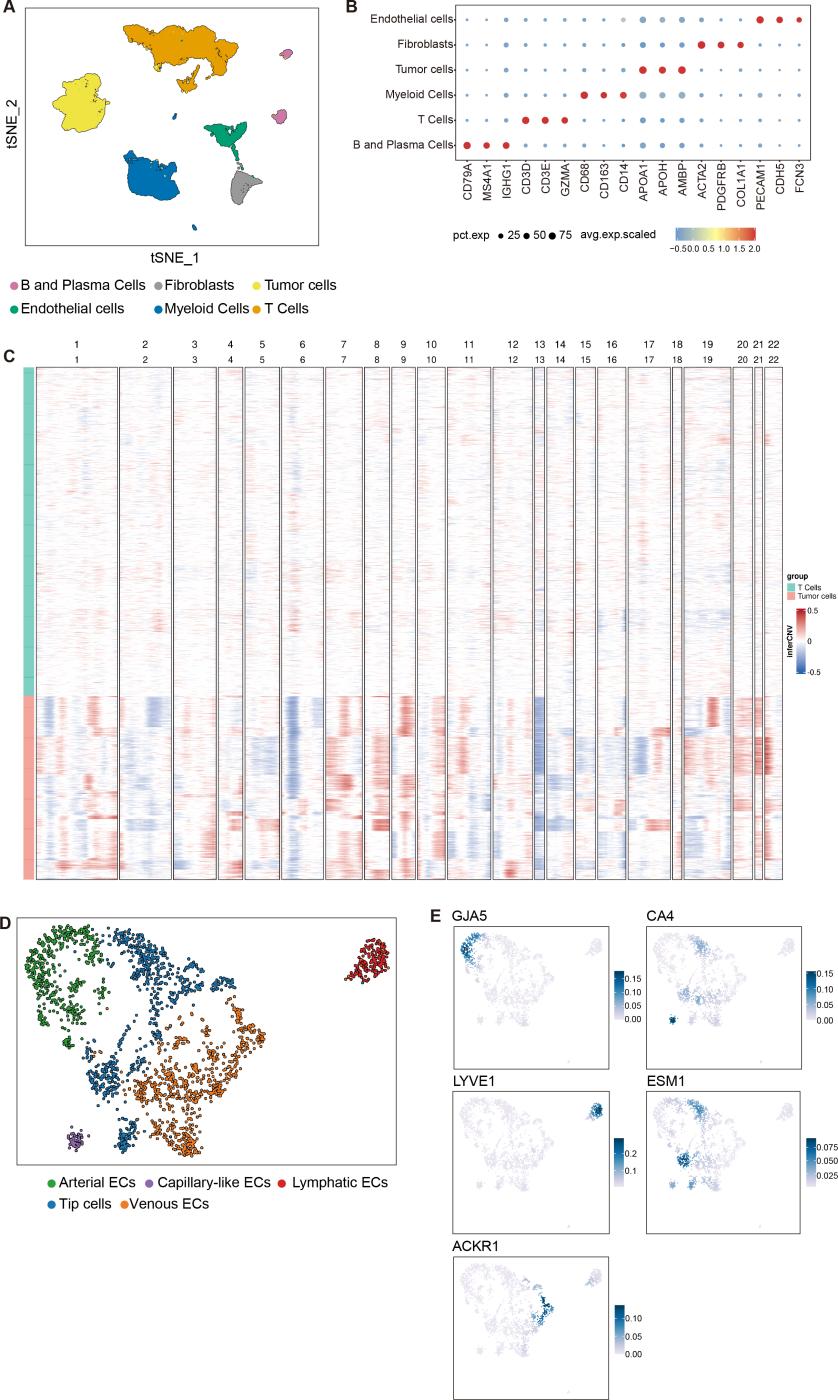
Identification of cell types in HCC tumor microenvironment revealed by single-cell analysis. **(A)** UMAP visualization showed distinct cell populations in the tumor microenvironment. Six major cell types were identified by distinct colors: Fibroblasts are shown in gray, T cells in orange, B and Plasma Cells in pink-purple, Endothelial cells in green, Myeloid Cells in blue, and Tumor cells in yellow. **(B)** Dot plot showing the average scaled expression (color intensity) and percentage of expressing cells (dot size) for canonical marker genes across annotated cell populations in the HCC microenvironment. Cell types include: B and plasma cells (*CD79A*, *MS4A1*, *IGHG1*), T cells (*CD3D*, *CD3E*, *GZMA*), myeloid cells (*CD68*, *CD163*, *CD14*), tumor cells (*APOA1*, *APOH*, *AMBP*), fibroblasts (*ACTA2*, *PDGFRB*, *COL1A1*), and endothelial cells (*PECAM1*, *CDH5*, *FCN3*). **(C)** Copy number variation (CNV) landscape of tumor cells compared to T cells, assessed by inferCNV. Each row represents a single cell; columns represent autosomes (chr1–22). The heatmap displays the interCNV score (log2 ratio of mean expression in test vs. reference cells), with red indicating copy number gains and blue indicating losses. **(D)** Higher resolution clustering of endothelial cell subtypes. Five distinct endothelial cell populations were identified: Arteries (green), Capillaries (purple), Lymphatics (red), Tip cells (dark blue), and Veins (orange). **(E)** Feature plots showing the expression of endothelial subtype-specific marker genes within the endothelial cell clusters. Expression of marker genes in the endothelial compartment is visualized using Nebulosa on the UMAP embedding. The color intensity (blue) represents kernel density estimates, indicating the relative spatial density of cells expressing each gene. Density values are unitless and derived from the distribution of cells in the embedding space; they are not gene expression values.

Cluster identities were assigned through an integrative approach: (1) differentially expressed genes (DEGs) for each cluster were identified using the FindAllMarkers function (log_2_ fold-change > 0.25, adjusted *p* < 0.05, minimum fraction of expressing cells = 0.1); (2) top DEGs were compared against established endothelial lineage signatures from the pan-cancer endothelial atlas by Li et al (15), which defines four major tumor-associated endothelial states—arterial (*GJA5*), capillary (*CA4*), Lymphatics (*LYVE1*), tip (*ESM1*), and venous (*ACKR1*)—based on conserved transcriptional programs across human cancers. Gene expression overlays in **Figures 1C, E** were generated using the Nebulosa R package (version 1.0.1) to visualize spatial expression patterns based on normalized log-counts.

### Pseudotime trajectory inference

We reconstructed endothelial differentiation trajectories using two complementary algorithms: Monocle3 (v1.3.4) and Slingshot (v2.7.0). For Monocle3, we first converted the Seurat object into a new_cell_data_set using the as.cell_data_set() function, followed by dimensionality reduction via UMAP. Based on prior biological knowledge (7), we manually designated VenECs—identified by high expression of *ACKR1* and *NR2F2*—as the root population using the order_cells function.

For Slingshot, we provided the same UMAP embedding and cluster annotations as input to the slingshot() function without specifying any start or end clusters, allowing the algorithm to infer lineage topology in a fully data-driven manner.

### TCGA integrated survival analysis using the 30-gene signature

For survival stratification, we computed a 30-gene signature score for each patient by taking the mean Z-score-normalized expression of the 30 genes in the harmonized matrix. Patients were then dichotomized into high- and low-expression groups using the median signature score as the cutoff. Kaplan–Meier survival analysis was performed to compare overall survival between the two groups.

### Construction of the VERS score prediction model

We began by performing univariate Cox proportional hazards regression to screen for vein endothelial-related risk scores (VERS) genes associated with patient prognosis. To ensure comprehensive inclusion of potentially relevant candidates, we adopted a lenient significance threshold of *p* < 0.1 at this stage. Next, genes passing this initial filter were subjected to multivariate Cox regression analysis, with statistical significance defined at *p* < 0.05, to identify an independent set of prognostic VERS genes.

To refine the model and mitigate overfitting, we applied least absolute shrinkage and selection operator (LASSO) regularization using the glmnet R package (version 4.1-7). This penalized regression approach selected the most parsimonious gene signature while optimizing predictive performance. The final VERS score for each patient was calculated using the following linear combination:


$$VERS\;score=\;\sum_{i=1}^{n}Coef(\beta i)*Exp(Xi)$$

where βi denotes the regression coefficient derived from the LASSO-Cox model for the *i*-th gene, Exp(Xi) represents its normalized expression level, and *n* is the total number of genes retained in the final signature.

For consistent risk stratification within each cohort, the median VERS score of that specific cohort was used as the cutoff to classify patients into high- versus low-VERS groups. This cohort-specific thresholding strategy prevents data leakage across datasets and ensures internal validity.

Kaplan–Meier survival curves were generated using the survival (version 3.5-7) and survminer (version 0.4.9) R packages to evaluate the association between VERS stratification and overall survival (OS). Additionally, time-dependent receiver operating characteristic (ROC) curves were constructed to assess the prognostic accuracy of the VERS score.

### Estimation of drug sensitivity (IC_50_) using oncoPredict

Drug sensitivity was inferred using the oncoPredict R package (version 1.2), a transfer learning framework that predicts drug response in tumor samples based on large-scale pharmacogenomic reference data. We used the Genomics of Drug Sensitivity in Cancer (GDSC2) dataset as the training reference, which includes experimentally measured IC_50_ values for over 200 compounds across ∼1,000 cancer cell lines. The TCGA tumor expression matrix (TPM values) was first log_2_-transformed after adding a pseudocount of 1. Only genes present in both the TCGA and GDSC2 datasets were retained, and the bottom 20% of low-variance genes were removed to reduce noise. Batch effects between the bulk RNA-seq (TCGA) and microarray (GDSC2) platforms were corrected using the empirical Bayes method implemented in oncoPredict (batchCorrect = “eb”). Drug response phenotypes from GDSC2 were power-transformed to approximate normality, and the calcPhenotype() function was applied to generate predicted IC_50_-like scores for each sample and drug. Higher predicted scores indicate greater resistance to the corresponding agent.

### Cell lines

The human HCC cell lines of Huh7 was acquired from Cell Bank, Chinese Academy of Sciences (Shanghai, China). Huh7 was cultured in Dulbecco’s modified Eagle’s medium (DMEM) (PM150210, Procell, China) containing 10% fetal bovine serum (164210-50, Procell, China), 1% streptomycin and penicillin (10378016, Gibco, USA). Human Umbilical Vein Endothelial Cells (HUVECs, Cat.#8000, ScienCell, USA) were cultured in Endothelial Cell Medium (Cat.#1001, ScienCell, USA) with additional 10% FBS, endothelial cell growth supplement and 1% streptomycin and penicillin. All cell lines were cultured in a humidified atmosphere with 5% CO2 at 37°C.

### Mouse studies

Seven-week-old male B-NDG mice were purchased from BesTest (Zhuhai, China) and maintained in a specific pathogen-free (SPF) facility. For subcutaneous xenograft modeling, mice were injected in the upper left flank with 20 μL of a cell suspension containing either 2 × 10^6^ Huh7 cells alone, or a mixture of 2 × 10^6^ Huh7 cells and 1 × 10^6^ HUVECs transduced with either shMARCKS or a non-specific shRNA control. Tumor growth was assessed by measuring the length and width of the tumor masses at the inoculation site using calipers. The tumor volumes were calculated by using the formula: volume (mm (3)) = π/6 × length × width × width. After 6 weeks, the tumor-bearing mice were euthanized. Tumors were excised, fixed in 4% paraformaldehyde for histopathological analysis, and snap-frozen in liquid nitrogen for subsequent gene expression studies. Mice were euthanized by gradual-fill CO_2_ inhalation at a displacement rate of 20% of the chamber volume per minute, followed by cervical dislocation to ensure death.

### Statistical analysis

Data were analyzed using GraphPad Prism 9.0 and presented as mean ± SEM. Statistical comparisons between two groups were performed using a two-tailed Student’s t-test. For multiple group comparisons, one-way ANOVA followed by *post hoc* analysis was used. A *p*-value < 0.05 was considered statistically significant. Specific sample sizes (n) and *p*-values are provided in the figure legends.

## Results

### Single-cell analysis reveals the cellular composition of HCC and characterizes endothelial cell subpopulations

To dissect the cellular landscape of HCC, we integrated three scRNA-seq datasets of HCC tumors (GSE151530, GSE125449 and GSE149614) from the GEO database. After batch effect correction, a total of 41,301 cells were retained for downstream analysis. Dimensionality reduction using UMAP identified six major cell populations: T cells, B cells, myeloid cells, endothelial cells, tumor cells, and fibroblasts (**Figure 1A**). We employed unsupervised clustering and marker-based annotation to delineate the cellular architecture of the HCC microenvironment at single-cell resolution. Tumor cells were identified by high expression of hepatocyte-derived apolipoproteins, including *APOA1*, *APOH*, and *AMBP*—a signature consistently observed in malignant hepatocytes across multiple HCC scRNA-seq cohorts (16). Immune cells were broadly defined by *PTPRC* (*CD45*) expression and further subclassified into B and plasma cells (*CD79A*, *MS4A1*, *IGHG1*), T cells (*CD3D*, *CD3E*, *GZMA*), and myeloid cells (*CD68*, *CD163*, *CD14*). Stromal compartments comprised fibroblasts (*ACTA2*, *PDGFRB*, *COL1A1*) and endothelial cells (*PECAM1*, *CDH5*, *FCN3*), collectively reconstructing the major lineages present in the HCC ecosystem (**Figure 1B**). We further validated the annotation of tumor cells using inferCNV-based CNV analysis. Compared to T cells, tumor cells exhibited widespread chromosomal alterations, supporting the malignant nature of the annotated tumor cell population. In contrast, T cells displayed near-diploid profiles with minimal CNV signals, confirming their non-malignant origin (**Figure 1C**). Given the critical role of angiogenesis in tumor progression, we further focused on tumor-associated endothelial cells to delineate the tumor vasculature microenvironment (TVM). Endothelial cells were subclustered into LECs (*LYVE1*) and vascular endothelial cells. The latter were further stratified into four subpopulations: ArtECs (*GJA5*), CapECs (*CA4*), Tip cells (*ESM1*), and VenECs (*ACKR1*) (**Figures 1D, E**).

### Differentiation trajectory analysis of tumor-associated endothelial cells in HCC

**Figure 2:**
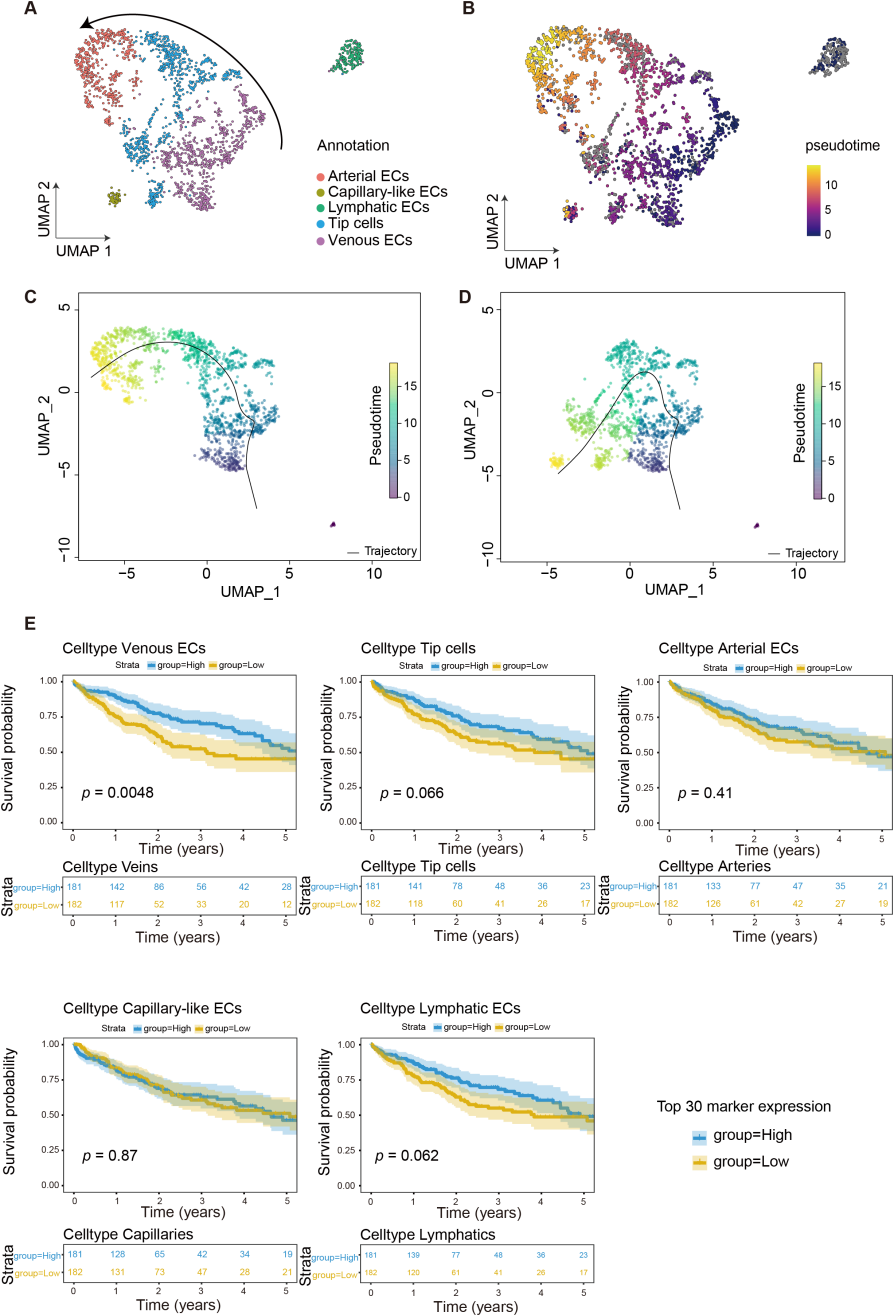
Endothelial cell heterogeneity and its clinical significance in OS. **(A)** UMAP visualization of endothelial cell subtypes identified five distinct populations: Arteries (pink), Capillaries (olivedrab), Lymphatics (aquamarine), Tip cells (blue), and Veins (purple). The curved arrow indicated a potential developmental or differentiation trajectory between cell types. **(B)** UMAP visualization of the same endothelial cell populations was colored by pseudotime analysis, with a gradient from yellow (late pseudotime, value 10) to dark blue (early pseudotime, value 0). **(C, D)** Inferred developmental trajectories of endothelial cells were visualized on UMAP plots. Black curves represented the predicted differentiation paths across the endothelial cell landscape, with cells colored by different developmental stages or states in each panel. **(E)** Kaplan–Meier survival analyzes showed the correlation between endothelial subtype-specific gene signatures and patient outcomes. Each survival plot included the number of patients at risk at different time points (0–5 years) for both high (blue) and low (yellow) expression groups. Analysis was based on the top 30 marker genes for each endothelial cell subtype.

Tumor angiogenesis involves sprouting from pre-existing vasculature to form new vessels (17, 18). To investigate the developmental trajectory of endothelial cells within HCC tumors, we performed pseudotime analysis using the “monocle3” R package based on transcriptional profiles (**Figures 2A, B**). Guided by prior biological knowledge of angiogenic sprouting, VenECs were manually designated as the root population in the Monocle3 analysis, from which the inferred trajectory branched into tip cells and ultimately culminated in ArtECs, consistent with previous reports (7). Independently, a parallel pseudotime analysis was performed using the “Slingshot” R package in an unsupervised manner, without predefined root specification. This analysis yielded a comparable trajectory structure, with VenECs positioned at the origin of the differentiation pathway and ArtECs representing the terminal state (**Figures 2C, D**).

To gain further insights into the regulatory programs governing ECs differentiation, we applied SCENIC to infer transcription factor activity across ECs subsets. Notably, VenECs exhibited specific activation of *SRF*, *ETS1*, *SMAD5*, *ETV7*, *SOX18*, and *TEF* (**Supplementary Figure 1A**), many of which have been implicated in angiogenesis. For instance, *SRF* regulates filopodia formation and cell contractility during endothelial sprouting (19), while *ETS1* promotes angiogenesis via the induction of pro-angiogenic factors such as *VEGF* and *HGF* (20). The transcriptional activity profile of VenECs underscores their high angiogenic potential and supports their role as progenitor cells in neovascularization. By contrast, tip cells showed enriched expression of *FOSB*, *JUN*, and members of the *KLF* family, while ArtECs exhibited increased expression of *ELF1*, *KLF2*, and *HES5*, transcription factors associated with vascular maturation and stability (**Supplementary Figures 1B–D**). *MAF* transcription factor is highly expressed in lymphatic vessels and preserves the differentiation status of lymphatic endothelial cells (21) (**Supplementary Figure 1E**).

To investigate the prognostic relevance of ECs subsets, we performed Kaplan–Meier survival analysis on HCC patients from the TCGA dataset. Patients were stratified into high and low expression groups based on the median expression of the top 30 marker genes for each ECs subset. Notably, elevated expression of VenECs signature genes was significantly associated with poorer overall survival, whereas gene signatures for tip cells, ArtECs, CapECs, and LECs showed no significant correlation with prognosis (**Figure 2E**), suggesting a more limited role of these subtypes in determining clinical outcome in HCC.

### A VenECs-derived gene signature predicted prognosis, immune infiltration and drug sensitivity in HCC patients

To evaluate the prognostic relevance of gene expression signatures derived from VenECs, we performed LASSO Cox regression analysis in the training cohort using the top 30 highly expressed genes within the VenECs subpopulation, with the corresponding coefficient values summarized in **Table 1**. This analysis identified five genes—*MARCKS*, *IGFBP4*, *IGFBP5*, *RAMP3*, and *TFF3*, that were significantly associated with overall survival.

**Table 1:** LASSO-selected genes associated with overall survival and corresponding coefficients.

Gene	Coef
*MARCKS*	0.00317540375022098
*IGFBP5*	0.0006765471206883
*RAMP3*	-0.008689366149794620
*TFF3*	-0.000040330373572239
*IGFBP4*	-0.000044310590968314

**Figure 3:**
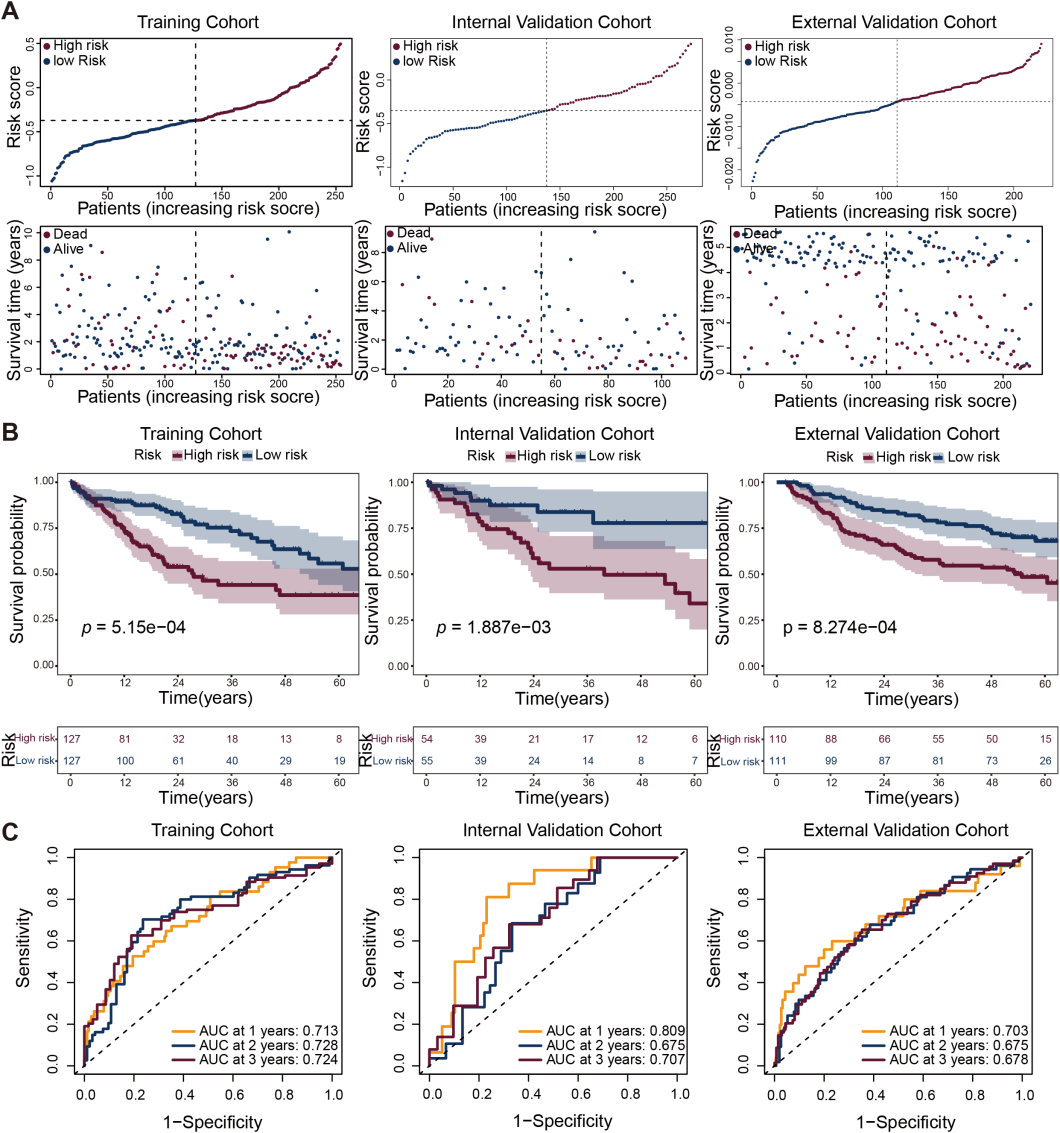
Validation of the prognostic risk stratification model across multiple cohorts. **(A)** Distribution of risk scores and associated survival outcomes. Upper panels showed the risk score distribution with patients arranged by increasing risk score, stratified into low-risk (dark blue) and high-risk (dark red) groups based on the optimal cutoff threshold (vertical dashed line) in training, internal validation, and external validation cohorts. Lower panels displayed corresponding patient survival time, with alive patients (dark blue dots) and deceased patients (dark red dots) plotted against their risk scores. **(B)** Kaplan–Meier survival curves compared high-risk (dark red) and low-risk (dark blue) patient groups across all three cohorts. **(C)** Receiver operating characteristic (ROC) curves evaluated model performance at 1 year (orange), 2 years (dark blue), and 3 years (dark red) across all cohorts.

Based on the expression levels of these five genes, the VERS model was constructed. Patients were stratified into high-risk and low-risk groups according to the median VERS value in the training cohort, internal validation cohort, and external validation cohort. Across all cohorts, patients in the high-risk group exhibited significantly worse prognosis compared to those in the low-risk group (**Figure 3A**). Kaplan–Meier survival analysis demonstrated that high-risk patients had markedly reduced overall survival (OS) in the training cohort (*p* = 5.15 × 10^-4^), a finding that was consistently validated in the internal (*p* = 1.887 × 10^-3^) and external (*p* = 8.274 × 10^-4^) cohorts (**Figure 3B**).

The predictive performance of the VERS model was further assessed using ROC curve analysis. The area under the curve (AUC) values for 1, 2 and 3-year OS in the training cohort were 0.713, 0.728, and 0.724, respectively. The corresponding AUCs in the internal validation cohort were 0.809, 0.675, and 0.707, and in the external cohort were 0.703, 0.675, and 0.678 (**Figure 3C**). These results indicate that the VERS model demonstrates robust prognostic power across independent datasets.

**Figure 4:**
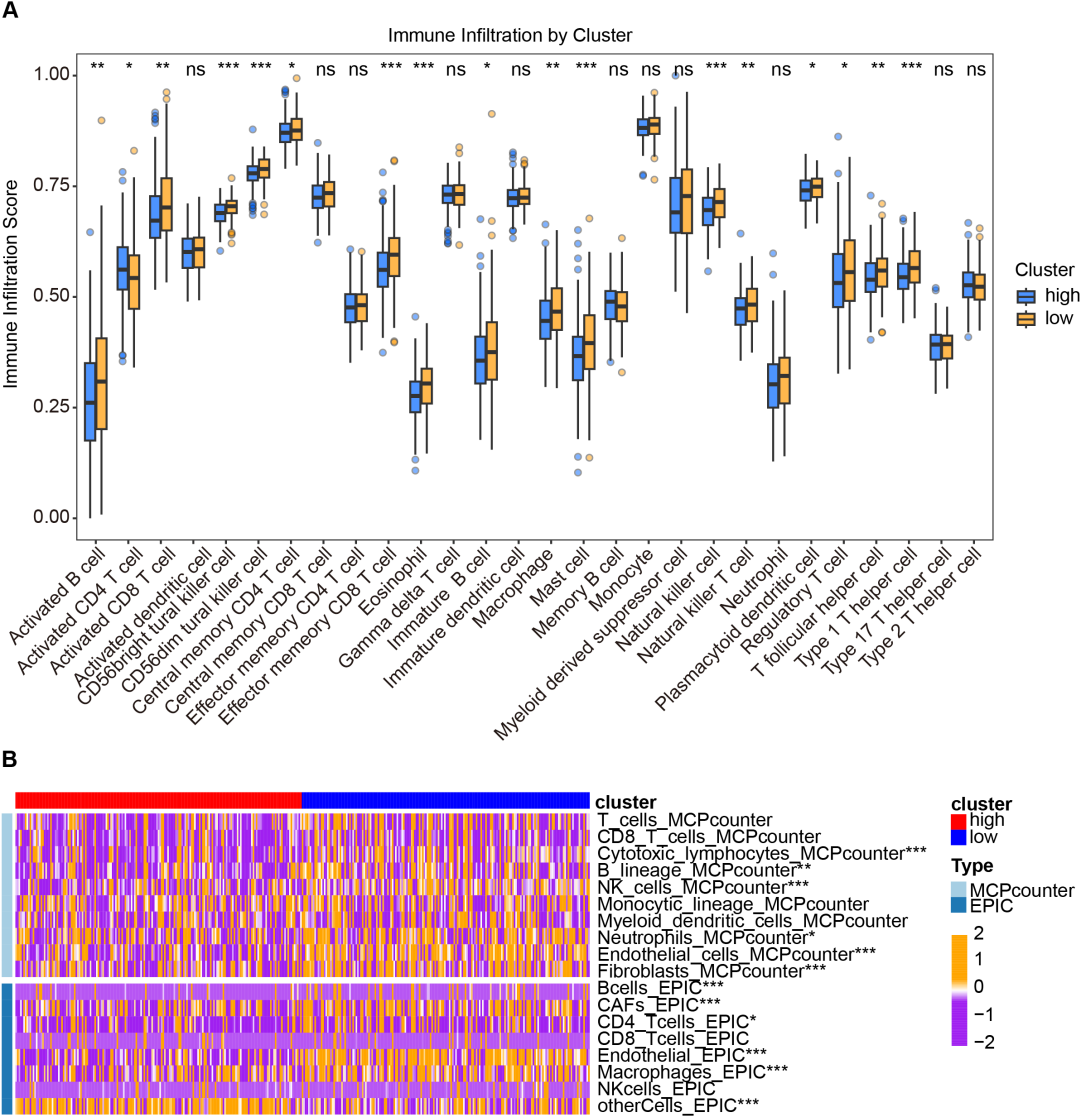
Comparison of immune infiltration characteristics between low-risk and high-risk groups. **(A)** Immune infiltration levels of 28 immune cell subtypes were compared between the high and low clusters using single-sample gene set enrichment analysis (ssGSEA) (low-risk group n= 184; high-risk group n= 185). **(B)** Heatmap depicting immune and stromal cell infiltration estimated by MCPcounter and EPIC, across the high and low clusters. The heatmap uses a purple-to-orange color gradient to represent standardized z-scores of immune cell abundance, with purple indicating below-average levels (negative z-scores), white representing average levels (z-score = 0), and orange indicating above-average levels (positive z-scores). Samples are grouped into high-risk (red annotation) and low-risk (blue annotation) clusters based on the previously defined risk score. Values are z-score normalized within each immune cell type and represent relative enrichment rather than absolute immune infiltration levels. Data are presented as mean ± SEM, and statistical significance was determined by one-way ANOVA or Student’s t-test; ns *P>*0.05, *P < 0.05, **P < 0.01, ***P < 0.001.

Given the critical role of the tumor immune microenvironment (TIME) in shaping patient prognosis and therapeutic response, we next explored the association between the VERS signature and immune characteristics. Specifically, we investigated whether differences in immune cell infiltration could underlie the distinct clinical outcomes observed between the high- and low-risk groups. To this end, comprehensive immune infiltration analyzes using multiple computational algorithms were performed. As shown in **Figure 4**, ssGSEA revealed significantly decreased infiltration of various immune effector cells, including activated B and CD8 T cells, CD56dim tural killer cell, central memory CD4 T cell, effect memory CD8 T cell, macrophages, naturalc killer cell and so on, in the high cluster (**Figure 4A**). Furthermore, cross-validation with multiple deconvolution methods (MCPcounter, EPIC) confirmed that the low cluster was characterized by a more immune-enriched microenvironment, whereas the high cluster showed a relatively immune-desert phenotype (**Figure 4B**). These findings suggest that the VenECs-related gene signature may serve as a potential biomarker for evaluating the immune landscape and predicting clinical outcomes in HCC patients.

**Figure 5:**
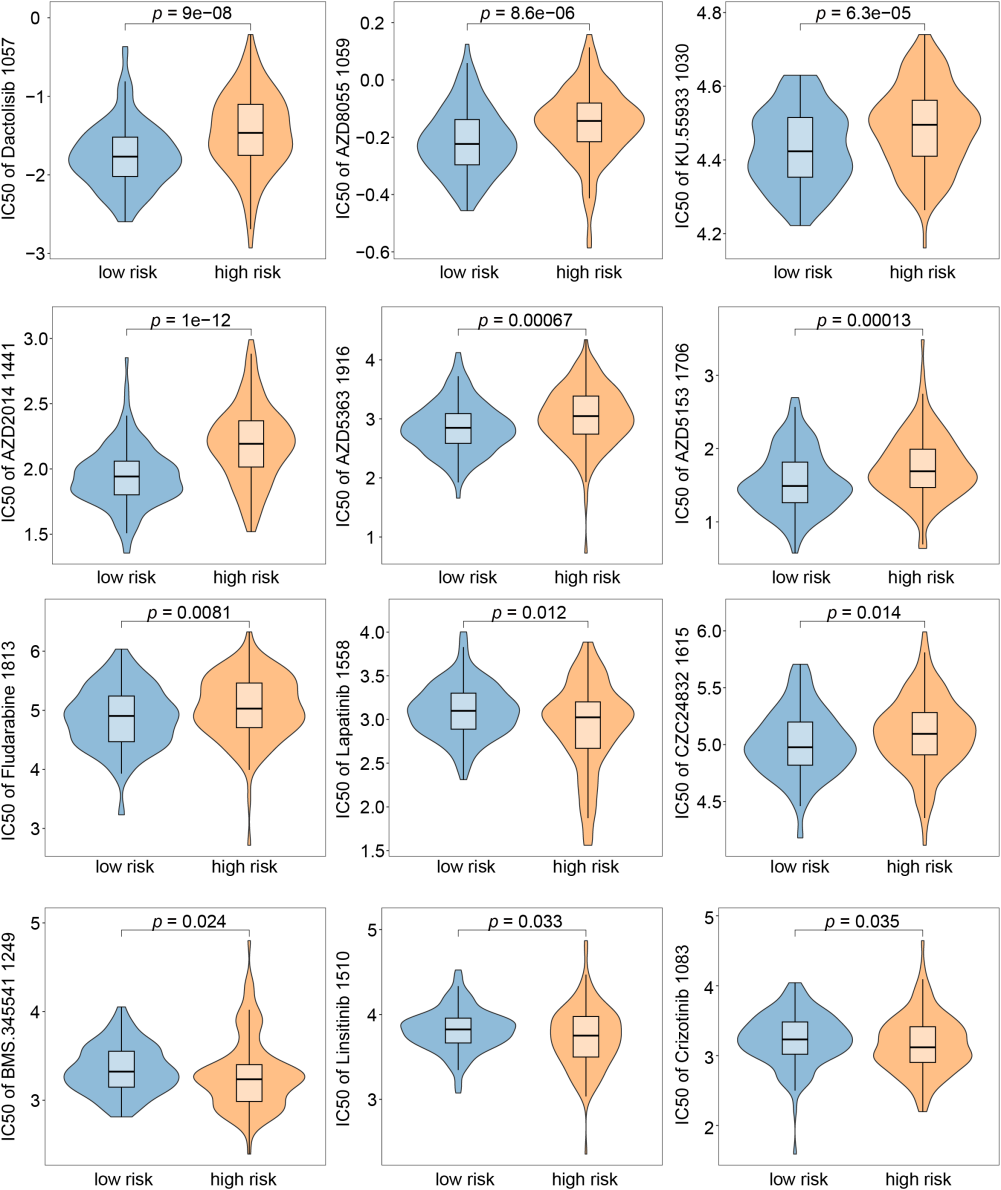
Drug sensitivity differences between low-risk and high-risk groups. Violin plots illustrated the estimated half-maximal inhibitory concentration (IC50) values for 12 anti-cancer drugs in low-risk (blue) and high-risk (orange) patient groups.

To explore the clinical utility of the model in guiding therapeutic strategies, we further assessed drug sensitivity between the high-risk and low-risk groups. High-risk patients exhibited significantly higher estimated IC50 values for multiple therapeutic agents, suggesting reduced drug responsiveness (**Figure 5**). Collectively, our findings indicate that the VERS model has potential utility as a prognostic tool across diverse patient populations and may offer insights into treatment sensitivity in HCC.

### High *MARCKS* expression is significantly associated with poor prognosis in HCC patients

**Figure 6:**
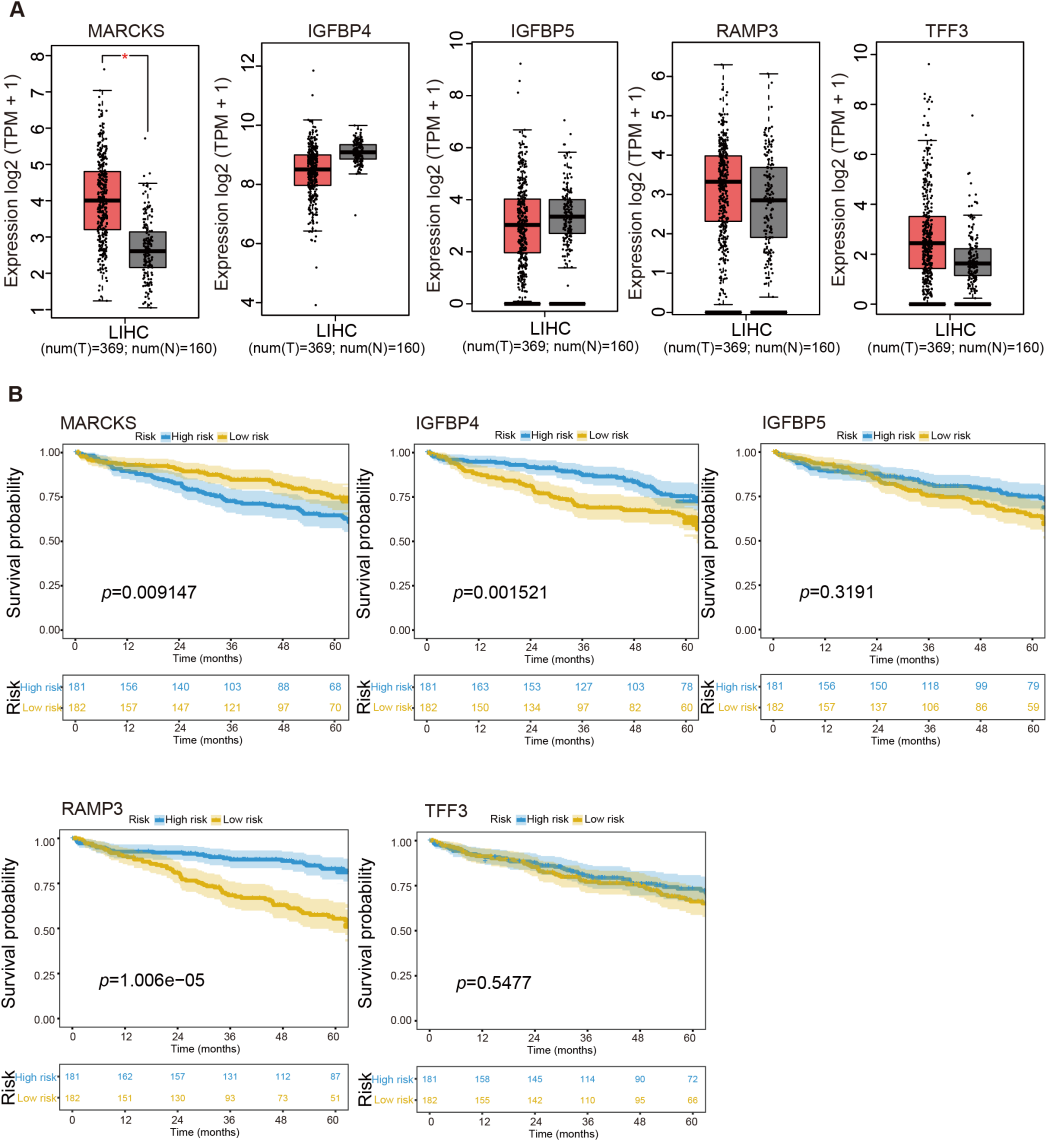
Expression of key biomarkers and survival analysis in LIHC. Boxplots showed the log2 expression (TPM + 1) of **(A)** *MARCKS*, *IGFBP4*, IGFBP5, *RAMP3* and *TFF3* in liver hepatocellular carcinoma (LIHC) samples (num (T) = 369; num (N) = 160). The expression levels were shown for both tumor and normal tissues, with the comparison between groups indicated. **(B)** Kaplan–Meier survival analysis was performed for the indicated genes in LIHC. Survival curves were shown for *MARCKS*, *IGFBP4*, *IGFBP5*, *RAMP3* and *TFF3*, comparing high-risk and low-risk groups based on expression levels. The number of individuals at risk in each group at selected time points was displayed at the bottom of each survival plot. Data are presented as mean ± SD, and statistical significance was determined by Student's t-test; ns P>0.05, *P < 0.05.

To elucidate the biological relevance of the VERS model, we performed a comprehensive analysis of the five prognostic genes identified. Gene expression profiles from TCGA and GTEx databases revealed that only *MARCKS* was significantly upregulated in tumor tissues compared to adjacent normal tissues (**Figure 6A**). In contrast, *IGFBP4*, *IGFBP5*, *RAMP3*, and *TFF3* showed no statistically significant differences in expression between tumor and non-tumor tissues (**Figure 6A**).

Kaplan–Meier survival analyzes stratified patients into high- and low-expression groups based on the expression levels of each gene. Notably, only patients with high *MARCKS* expression exhibited significantly worse overall survival compared to those with low expression (*p* = 0.009147; **Figure 6B**), suggesting that *MARCKS* may be a key driver of unfavorable prognosis in HCC. These findings indicate that the *MARCKS* gene has the potential to be used as a prognostic biomarker in HCC.

### Endothelial-associated *MARCKS* promotes HCC progression *in vitro*

**Figure 7:**
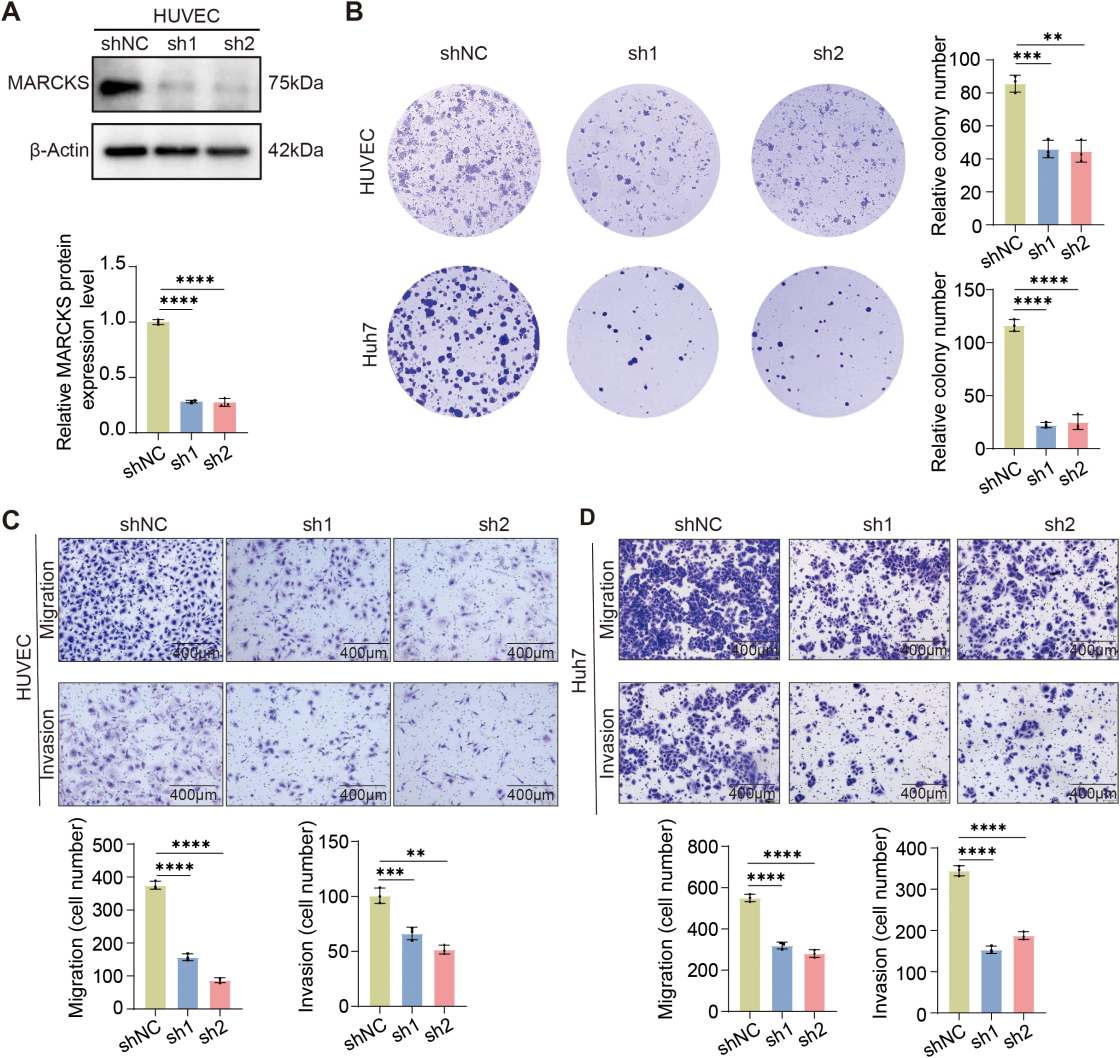
The effect of *MARCKS* knockdown in endothelial cells on HUVEC and HCC cell behavior. **(A)** Western blot analysis of *MARCKS* protein expression in HUVEC cells infected with shNC, sh1, or sh2 shRNA constructs. β-Actin was used as the loading control. **(B)** Colony formation assay showed the effects of *MARCKS* knockdown in HUVEC cells (upper panel) and Huh7 cells co-cultured with *MARCKS* knockdown HUVEC conditioned medium (lower panel) (n = 3 per group). **(C)** Migration and invasion assays in HUVEC cells after *MARCKS* knockdown. Representative images of migration and invasion (upper panels) and bar graphs quantified the migration and invasion abilities (lower panels) (n = 3 per group). **(D)** Migration and invasion assays in Huh7 cells co-cultured with conditioned medium from *MARCKS* knockdown HUVEC cells. Representative images of migration and invasion (upper panels) and bar graphs quantified the migration and invasion abilities (lower panels) (n = 3 per group). Data are presented as mean ± SD, and statistical significance was determined by one-way ANOVA or Student’s t-test; ns *P >*0.05, **P < 0.01, ***P < 0.001, ****P < 0.0001.

As the VERS model—constructed from the expression levels of five genes (*MARCKS*, *IGFBP4*, *IGFBP5*, *RAMP3*, and *TFF3*)—was established for inter-patient risk stratification, *MARCKS* was selected as a biologically tractable candidate for functional validation, given its dominant contribution to the risk score, its specific upregulation in tumor endothelial cells, and its established relevance to endothelial cell function. To investigate the functional implications of endothelial *MARCKS* expression in HCC progression, we generated *MARCKS* knockdown HUVEC cells using a lentivirus expressing *MARCKS*-targeting shRNA. Efficient knockdown of *MARCKS* was confirmed at the protein level (**Figure 7A**). Functional assays demonstrated that *MARCKS* silencing significantly impaired HUVEC clonogenicity, migration, and invasion abilities (**Figures 7B**, upper panel; **Figure 7C**).

To further evaluate the influence of endothelial *MARCKS* on tumor cell behavior, conditioned media from *MARCKS*-silenced HUVECs were used to co-culture Huh7 human hepatocellular carcinoma cells. Remarkably, Huh7 cells exposed to the supernatant of *MARCKS*-deficient HUVECs exhibited significantly reduced clonogenic, migratory, and invasive capacities (**Figures 7B**, lower panel; **Figure 7D**). These findings indicate that endothelial-associated *MARCKS* promotes pro-tumorigenic processes in HCC.

### Endothelial-associated *MARCKS* facilitates HCC tumor growth *in vivo*

**Figure 8:**
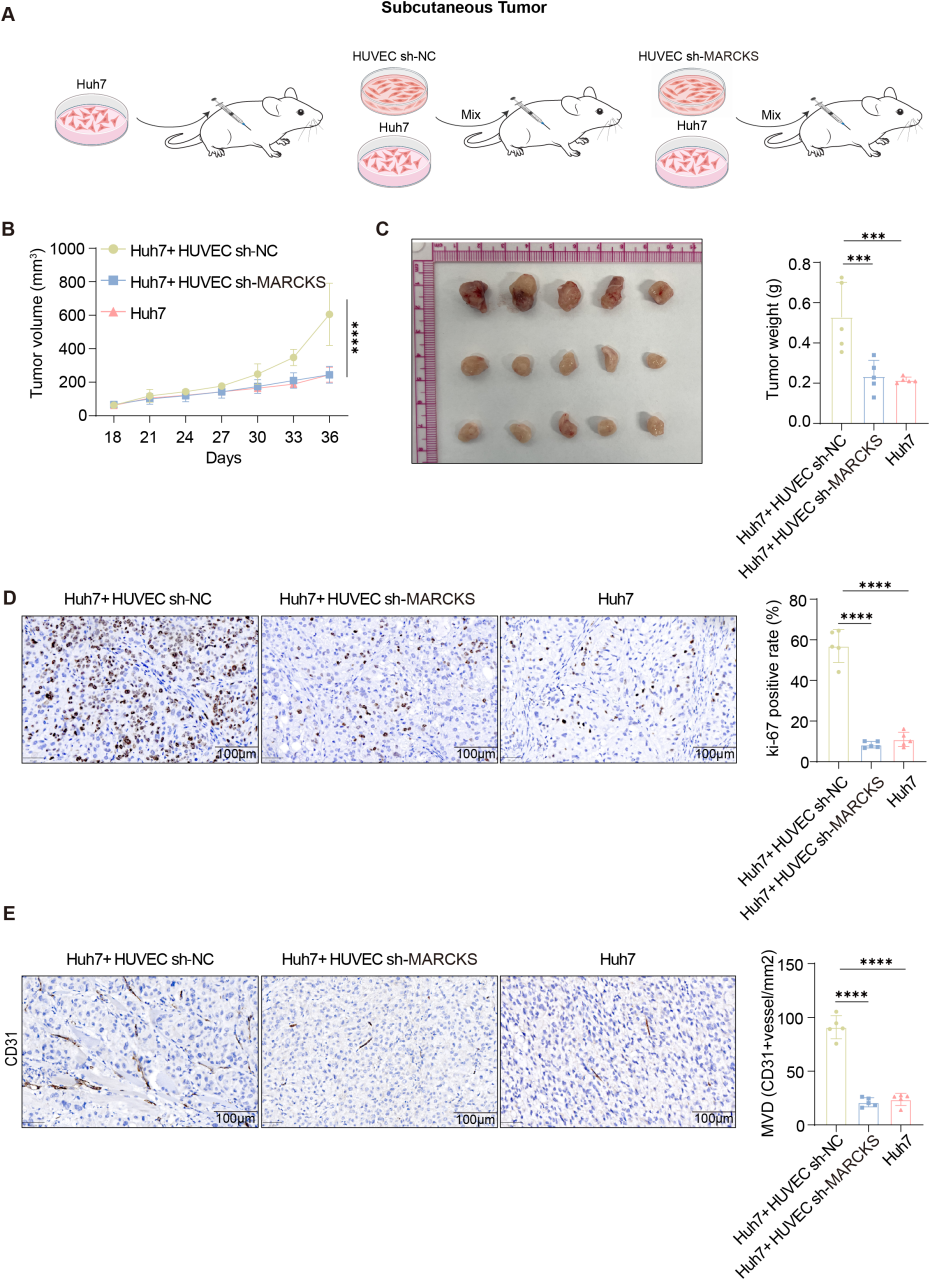
*MARCKS* knockdown in HUVECs suppresses Huh7 tumor growth *in vivo*. **(A)** Schematic illustration of subcutaneous tumor model establishment by mixing Huh7 cells with HUVEC sh-NC or HUVEC sh-*MARCKS* before injection into nude mice (n = 5 per group). **(B)** Tumor growth curves of Huh7 xenografts in different groups (n = 5 per group). **(C)** Representative images of harvested tumors and corresponding tumor weights (n = 5 per group). **(D)** Representative IHC staining of Ki-67 in xenograft tumors and quantification of Ki-67 positive rate. **(E)** Representative IHC staining of CD31 and quantification of microvessel density (MVD) (n = 5 per group). Scale bars: 100 μm. Data are presented as mean ± SD, and statistical significance was determined by one-way ANOVA or Student’s t-test; ns P>0.05, ***P < 0.001, ****P < 0.0001.

To corroborate our *in vitro* findings, we established a subcutaneous HCC xenograft model (**Figure 8A**). Co-injection of Huh7 cells with *MARCKS*-knockdown HUVECs (HUVEC-sh*MARCKS*) resulted in significantly attenuated tumor growth compared to co-injection with control HUVECs (HUVEC-shNC), while tumors in the Huh7-only group grew at an intermediate rate (**Figures 8B, C**).

Consistent with reduced tumor growth, Ki-67 staining revealed lower proliferative activity in tumors from the HUVEC-sh*MARCKS* group (**Figure 8D**). To examine the impact of *MARCKS* knockdown on tumor vasculature, we performed immunohistochemistry for CD31, an established endothelial marker. Tumors from HUVEC-sh*MARCKS* mice displayed reduced CD31-positive microvessel density, indicating diminished angiogenesis (**Figure 8E**).

Collectively, these data demonstrate that endothelial *MARCKS* plays a critical role in modulating the tumor microenvironment by promoting both tumor cell proliferation and neovascularization, underscoring its potential as a therapeutic target in HCC.

## Discussion

For advanced HCC patients ineligible for surgical resection, multi-kinase inhibitors such as sorafenib and lenvatinib provide only modest survival benefits due to acquired resistance and adverse effects (22–25), highlighting the need for more effective vascular-targeted therapies. Although recent studies have functionally characterized endothelial, lymphatic endothelial, and perivascular subpopulations across different angiogenic stages (26), the therapeutic potential of targeting the initiation of tumor angiogenesis remains largely unexplored.

In this study, we leveraged single-cell transcriptomic profiling to provide novel insights into the heterogeneity of endothelial cells in the HCC microenvironment. We identified functionally distinct endothelial subpopulations and developed a venous endothelial-related gene signature-based prognostic model, the VERS Model, which demonstrated robust predictive performance across multiple independent cohorts, thereby offering a valuable tool for clinical decision-making. Notably, among the five VERS genes, *MARCKS* stood out as the sole candidate consistently overexpressed in HCC tumors and independently predictive of poor survival, thereby justifying its selection for mechanistic investigation.

Using venous endothelial, HCC cell lines, and murine models, we demonstrated that *MARCKS*-driven vascular remodeling promotes HCC initiation and progression, supporting *MARCKS* as a potential therapeutic target. Endothelial cells were classified into five clusters, with trajectory analyzes by Monocle3 and Slingshot identifying VenECs as the origin of differentiation, highlighting their central role in shaping tumor vasculature. VenECs were enriched for *ACKR1*, a TECs marker that modulates tumor inflammation, though its widespread expression limits its specificity as a therapeutic target (27–30). These results underscore the value of trajectory-informed prognostic models and the need to identify novel targetable endothelial markers.

Our study first linked VenEC-specific genes to HCC prognosis. Using LASSO regression, we identified a five-gene signature (*MARCKS*, *IGFBP4*, *IGFBP5*, *RAMP3*, *TFF3*) that stratified patients into high- and low-risk groups with significantly different overall survival across multiple cohorts, highlighting its utility as a prognostic tool and a basis for personalized therapy.

Drug sensitivity analyzes revealed striking differences in therapeutic responses between risk groups. For instance, high-risk patients exhibited significantly higher IC50 values for Dactolisib 1057, a dual PI3K/mTOR inhibitor known to impair HUVEC proliferation, induce apoptosis, and suppress HCC growth *in vitro* and *in vivo* (31–33). Conversely, low-risk patients demonstrated greater sensitivity (lower IC50) to AZD2014 (Vistusertib) and AZD5363 (Capivasertib), suggesting enhanced responsiveness to PI3K/AKT/mTOR pathway inhibition in this subgroup. Notably, Foretinib, another mTOR inhibitor, has shown efficacy in halting NAFLD-associated HCC progression (34). These findings supported the incorporation of endothelial-based risk models into clinical workflows to predict drug responsiveness, thereby guiding individualized treatment selection.

To further investigate the pathological role of *MARCKS*, we compared its expression in different liver regions of HCC patients. *MARCKS* expression was significantly elevated in tumor tissues compared to adjacent non-tumorous liver and correlated with poor prognosis. These results aligned with prior studies that implicated *MARCKS* in multiple malignancies, where it contributed to therapeutic resistance and metastasis (35–37). In triple-negative breast cancer, *MARCKS* activates the PI3K/AKT/mTOR pathway to promote chemoresistance and metastasis (38, 39). In HCC, *MARCKS* expression on tumor-associated macrophages is associated with immune infiltration and poor outcomes (40). Thus, *MARCKS* may serve as both a prognostic biomarker and a modulator of the HCC tumor microenvironment.

Functionally, *MARCKS* knockdown in HUVECs markedly reduced their clonogenicity, migration, and invasion. Conditioned media from *MARCKS*-silenced HUVECs also suppressed malignant phenotypes in co-cultured Huh7 cells, indicating a critical role of endothelial *MARCKS* in promoting HCC progression. Together, these results support a model in which *MARCKS* regulates HCC aggressiveness, at least in part, via angiocrine/paracrine signaling. Based on its reported involvement in PI(4,5) P2-dependent membrane–cytoskeleton dynamics (41, 42), it is plausible that *MARCKS* depletion influences endothelial polarity and vesicular trafficking, thereby altering the endothelial secretory program and potentially modulating multiple pro-tumorigenic angiocrine signals, consistent with the network-based nature of endothelial–tumor communication. *In vivo*, co-implantation of Huh7 cells with *MARCKS*-knockdown HUVECs yielded significantly smaller tumors with reduced Ki67 proliferation indices, further substantiating *MARCKS* as a key regulator of endothelial-driven tumorigenesis.

This study has limitations. Although the conditioned-medium assays support an endothelial-to-tumor paracrine contribution, we did not perform systematic profiling of cytokines/growth factors (or extracellular vesicle cargos) in shMARCKS versus control HUVEC supernatants. Future studies combining multiplex cytokine arrays and/or secretome proteomics with functional perturbation (e.g., neutralizing antibodies or receptor blockade) will be important to identify dominant *MARCKS*-dependent mediators and establish a causal factor–phenotype link.

Collectively, these findings highlight the complexity of the HCC microenvironment, the prognostic and therapeutic relevance of VenECs, and the potential of the VERS model for patient stratification and personalized therapy. *MARCKS* emerges as a promising target to enhance anti-angiogenic efficacy, warranting further mechanistic studies and clinical validation.

## Data Availability

The datasets presented in this study can be found in online repositories. The names of the repository/repositories and accession number(s) can be found in the article/Methods.

## Glossary

AATs: anti-angiogenic therapies
ECs: endothelial cells
ArtECs: arterial ECs
VenECs: venous ECs
CapECs: capillary-like ECs
LECs: lymphatic ECs
HCC: hepatocellular carcinoma
IHC: Immunohistochemistry
*MARCKS*: Myristoylated Alanine-Rich C Kinase Substrate
scRNA-seq: single-cell RNA sequencing
OS: overall survival
TME: tumor microenvironment
TVM: tumor vasculature microenvironment
TECs: tumor endothelial cells
TACE: transarterial chemoembolization
VERS: Vein Endothelial-related Risk Scores
*VEGFR*: Vascular endothelial growth factor receptor
