# Supplementary material for: Venous endothelial remodeling mediated by MARCKS promotes angiogenesis and tumor progression in hepatocellular carcinoma: insights from single-cell RNA sequencing
Source: Front Immunol. 2026 Mar 12;17:1734982. doi: 10.3389/fimmu.2026.1734982 (PMC13017896; doi:10.3389/fimmu.2026.1734982)
Supplement: Supplementary file 1 [file DataSheet1.docx]

**Supplementary Materials**

**Supplementary Figure**

**
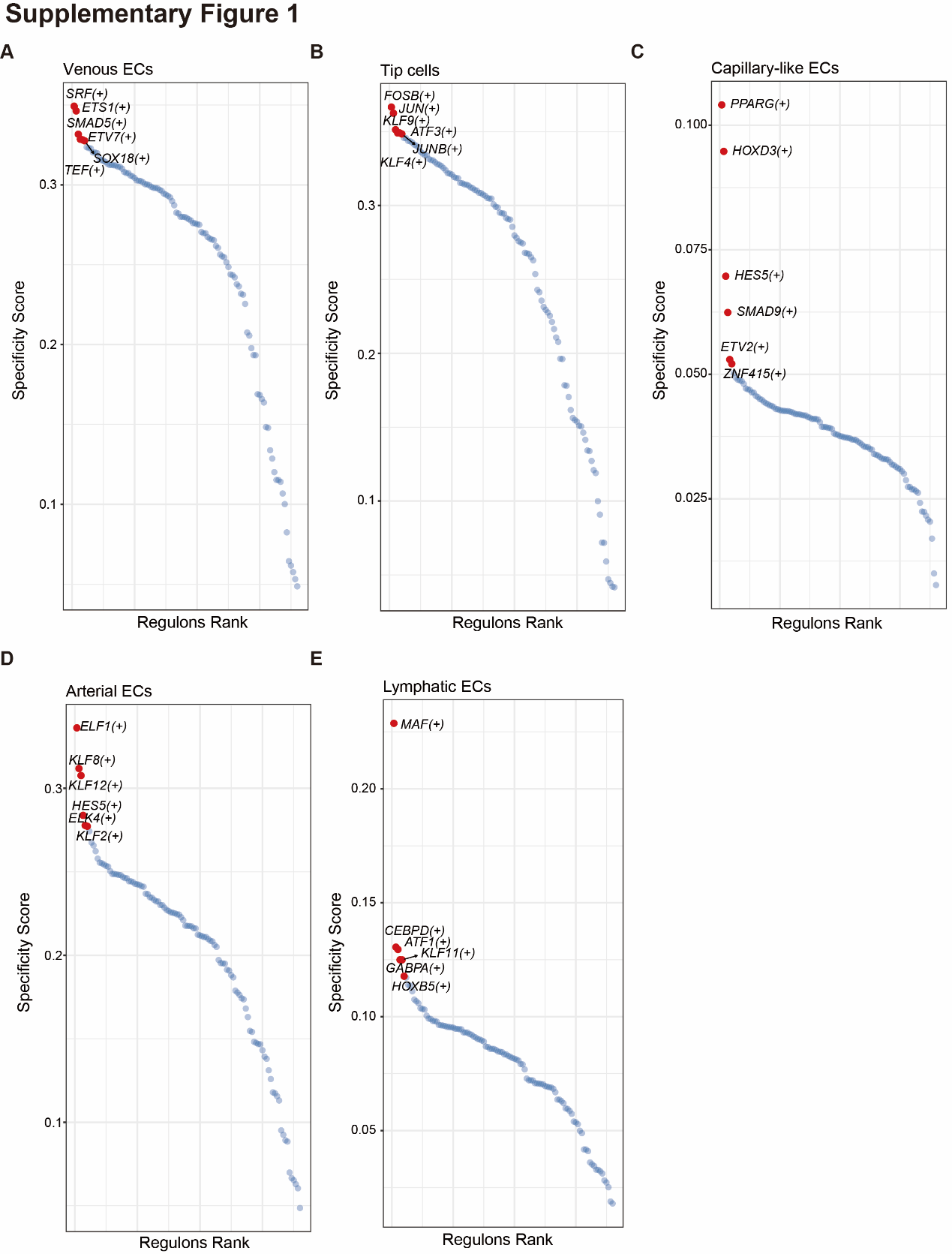
**

**Supplementary Figure 1: Transcription factor regulatory networks across endothelial cell subpopulations.** Ranked specificity score plots of transcription factor regulatory networks in different endothelial cell subtypes identified by SCENIC analysis. (A) Veins: Showed high specificity of *SRF*, *ETS1*, *SMAD5*, *ETV7*, *SOX18*, and *TEF* transcription factors. (B) Tip cells: Characterized by specific expression of *FOSB*, *JUN*, *KLF4*, and *JUNB*. (C) Capillaries: Displayed specificity for *PPARG*, *HOXD3*, *HES5*, *SMAD9*, *ETV3*, and *ZNF416* transcription factors. (D) Arteries: Enriched in *ELF1*, *KLF8*, *KLF19*, *HES5*, *ETS1*, and *KLF5* regulatory factors. (E) Lymphatics: Showed *MAF*, *CEBPB*, *ATF1*, *NFATC4*, *GATA5*, and *HOXB5* as key regulators. The x-axis represented regulon rank and the y-axis represented specificity score, with higher scores indicating greater specificity of transcription factor activity in each endothelial subtype. Red dots highlighted the most significant transcription factors that characterized each endothelial cell subpopulation's regulatory network.

**Supplementary Methods**

**Unsupervised Cell Clustering and Marker Gene Identification**

The scRNA-seq data analysis was conducted using the Seurat R package. Gene expression values were normalized using the "LogNormalize" method, which involved scaling the raw expression data by a factor of 10,000 and subsequently applying a logarithmic transformation. To identify features with significant variability, 2,000 highly variable genes were selected using the "FindVariableFeatures" function with the variance stabilizing transformation (VST) approach. Principal Component Analysis (PCA) was then performed on these highly variable genes. The number of principal components (PCs) used for downstream analysis was determined using an elbow plot, and in this study, 35 PCs were selected. To account for batch effects, Harmony (version 0.1.1) was applied with default parameters^1^.

For clustering, a graph-based approach was implemented using the shared nearest neighbor (SNN) and the Louvain algorithm. Specifically, the "FindNeighbors" and "FindClusters" functions in Seurat were used for this step. Visualization of clustering results was achieved using t-distributed Stochastic Neighbor Embedding (t-SNE) and Uniform Manifold Approximation and Projection (UMAP).

To delineate distinct cell clusters, differential gene expression analysis was performed using the "FindAllMarkers" function in Seurat. Differentially expressed genes were defined as those with a log fold-change greater than 0.25 and detectable expression in at least 25% of cells within either group being compared. Finally, cell clusters were annotated by comparing the expression profiles of known canonical marker genes. The marker genes for each cluster are described in detail in the main text of the article.

**Survival Analysis**

The TCGA samples were stratified into two groups ("low" and "high" expression groups) based on the median expression of the top 30 highly expressed genes identified for each of the 5 endothelial cell subclusters. Kaplan-Meier survival analysis was then performed to compare overall survival between the two groups for each subcluster. This was achieved by using the "survfit" and "Surv" functions from the "survival" R package (version 3.2.7) in combination with the ggsurvplot function from the "survminer" R package (version 0.4.3). The Kaplan-Meier curves were visualized with two strata corresponding to the "low" and "high" expression groups.

**ssGSEA Methodology**

To evaluate the differences in immune cell infiltration between different subtypes, we employed the single-sample Gene Set Enrichment Analysis (ssGSEA) method. This approach calculates an immune cell infiltration score for each sample, enabling the further analysis of how different subtypes influence the immune microenvironment. We performed the ssGSEA analysis using the R package GSVA (version 1.48.3)^2^.

**IOBR Methodology**

To evaluate the differences in immune cell infiltration between different risk subtypes, we employed the IOBR (Immuno-Oncology Biological Research) R package (version 1.48.3) for multi-omics immuno-oncology analysis. This tool integrates several immune microenvironment deconvolution algorithms, including MCPcounter and EPIC, allowing us to estimate the abundance of immune and stromal cells in the tumor microenvironment (TME) based on transcriptomic data^3^.

**Lentiviral Transduction of HUVECs**

HUVECs were transduced with lentiviral particles purchased from IGE Biotechnology Co., Ltd. (Guangzhou, China), carrying short hairpin RNAs (shRNAs) targeting *MARCKS*. Cells were seeded in T75 flasks and infected with the lentivirus according to the manufacturer’s instructions. After 6 hours of incubation, the medium was replaced with fresh complete endothelial cell medium. Stable cell lines were established by infecting HUVECs with lentiviruses encoding either shRNAs targeting *MARCKS* or a non-specific control sequence, followed by selection with puromycin. Two shRNA sequences targeting Homo sapiens MARCKS were used:

shMARCKS-1: 5′-GAATGGCCACGTGAAGGTAAACTCGAGTTTACCTTCACGTGGCCATTCTTTTTGAATT-3′

shMARCKS-2: 5′-GCGACCCGCAGGAGGCCAAGCCTCGAGGCTTGGCCTCCTGCGGGTCGCTTTTTGAATT-3′

**Collection of conditioned medium from HUVECs**

When HUVECs transduced with either shMARCKS or a non-specific shRNA control reached approximately 70% confluency in T75 flasks, the culture medium was replaced with 10 mL of complete endothelial cell medium supplemented with 10% fetal bovine serum (FBS), and the cells were incubated for an additional 24 hours.The collected supernatants were then concentrated, filtered through 0.2 μm membranes, and stored at -80℃ for subsequent use.

**Co-culture of Huh7 Cells with HUVEC-Conditioned Medium**

Huh7 cells were seeded in 6-well plates at a density of 2 × 10^5^ cells per well and allowed to adhere overnight. The following day, the culture medium was replaced with HUVEC-conditioned medium. HUVEC-conditioned medium from cells transduced with either shMARCKS or a non-specific shRNA control was used. Huh7 cells were incubated with the conditioned media for 48 hours.

**Colony Formation Assay**

HUVECs stably transduced with shMARCKS or a non-specific shRNA (shNC) were seeded into 6-well plates at 4,000 cells per well and cultured in complete endothelial medium. After 14 days, colonies were fixed with 4% paraformaldehyde, stained with 0.5% crystal violet, and counted under a microscope. Colonies with >50 cells were considered positive.

To assess the effect of HUVEC-derived factors on Huh7 cell proliferation, Huh7 cells were collected from **Co-culture of Huh7 Cells with HUVEC-Conditioned Medium** as described. Then the cells were then seeded at a consistent density into 6-well plates and cultured in fresh DMEM for 14 days. Colony formation was assessed as previously described. All experiments were conducted in triplicate and independently repeated at least three times.

**Migration and Invasion Assays**

Transwell migration and invasion assays were conducted to assess the motility of HUVECs and Huh7 cells. For HUVECs, cells stably expressing shMARCKS or a non-specific shRNA (shNC) were seeded into the upper chambers of 8.0 μm Transwell inserts (Corning) at 5 × 10^4^ cells per well in serum-free medium. For invasion assays, the inserts were pre-coated with Matrigel. The lower chambers were filled with complete endothelial medium containing 10% fetal bovine serum (FBS). After 24 hours of incubation at 37°C, migrated or invaded cells on the lower membrane surface were fixed, stained with crystal violet, and counted under a microscope.

Huh7 cells were collected from **Co-culture of Huh7 Cells with HUVEC-Conditioned Medium** as described.Then the cells were carefully collected and seeded into the upper chambers under similar conditions. The lower chambers contained DMEM with 10% FBS. After 48 hours, migrated or invaded Huh7 cells were processed as described above. All assays were performed in triplicate and repeated independently at least three times.

**Western Blot Analysis**

HUVECs transduced with shMARCKS or shNC were lysed in RIPA buffer with protease inhibitors. Equal protein amounts were separated by SDS-PAGE, transferred to PVDF membranes, blocked with 5% BSA in TBST, and incubated overnight at 4°C with antibodies against MARCKS and β-actin. After TBST washes, membranes were incubated with secondary antibody for 2 h at room temperature and detected using Omni-ECL ultra-sensitive chemiluminescence detection kit (SQ201, EpiZyme, USA). Band intensity was quantified with ImageJ.

**Immunohistochemistry (IHC) Staining**

Mouse tissues were fixed in 4% paraformaldehyde, paraffin-embedded, and sectioned (4 μm). After deparaffinization, rehydration, and heat-mediated antigen retrieval (EDTA solution, Beyotime), sections were incubated overnight at 4°C with primary antibodies against Ki67(1:1000, ab16667, Abcam), MARCKS (1:1000, 20661-1-AP, Proteintech), and CD31 (1:2000, ab182981, Abcam). Detection was performed using the Dako REAL EnVision Kit and DAB, followed by hematoxylin counterstaining. Positive cells were quantified in three random fields per section under a light microscope. Sections were counterstained with hematoxylin, dehydrated, and mounted for imaging. Positive staining was assessed by counting the number of positive cells in three random fields per section under a light microscope.

**References:**

1 Korsunsky, I. et al., Fast, sensitive and accurate integration of single-cell data with Harmony. NAT METHODS 16 1289 (2019).

2 Hanzelmann, S., Castelo, R. & Guinney, J., GSVA: gene set variation analysis for microarray and RNA-seq data. BMC BIOINFORMATICS 14 7 (2013).

3 Zeng, D. et al., IOBR: Multi-Omics Immuno-Oncology Biological Research to Decode Tumor Microenvironment and Signatures. FRONT IMMUNOL 12 687975 (2021).
